# Pharmacological characterization of crotamine effects on mice hind limb paralysis employing both *ex vivo* and *in vivo* assays: Insights into the involvement of voltage-gated ion channels in the crotamine action on skeletal muscles

**DOI:** 10.1371/journal.pntd.0006700

**Published:** 2018-08-06

**Authors:** Sunamita de Carvalho Lima, Lucas de Carvalho Porta, Álvaro da Costa Lima, Joana D’Arc Campeiro, Ywlliane Meurer, Nathália Bernardes Teixeira, Thiago Duarte, Eduardo Brandt Oliveira, Gisele Picolo, Rosely Oliveira Godinho, Regina Helena Silva, Mirian Akemi Furuie Hayashi

**Affiliations:** 1 Departamento de Farmacologia, Escola Paulista de Medicina (EPM), Universidade Federal de São Paulo (UNIFESP), São Paulo, Brazil; 2 Departamento de Fisiologia, Universidade Federal do Rio Grande do Norte (UFRN), Natal, Brazil; 3 Laboratório Especial de Dor e Sinalização, Instituto Butantan, São Paulo, Brazil; 4 Departamento de Bioquímica e Imunologia, Universidade de São Paulo (USP-RP), Ribeirão Preto, Brazil; Instituto de Biomedicina de Valencia, SPAIN

## Abstract

The high medical importance of *Crotalus* snakes is unquestionable, as this genus is the second in frequency of ophidian accidents in many countries, including Brazil. With a relative less complex composition compared to other genera venoms, as those from the *Bothrops* genus, the *Crotalus* genus venom from South America is composed basically by the neurotoxin crotoxin (a phospholipase A2), the thrombin-like gyroxin (a serinoprotease), a very potent aggregating protein convulxin, and a myotoxic polypeptide named crotamine. Interestingly not all *Crotalus* snakes express crotamine, which was first described in early 50s due to its ability to immobilize animal hind limbs, contributing therefore to the physical immobilization of preys and representing an important advantage for the envenoming efficacy, and consequently, for the feeding and survival of these snakes in nature. Representing about 10–25% of the dry weight of the crude venom of crotamine-positive rattlesnakes, the polypeptide crotamine is also suggested to be of importance for antivenom therapy, although the contribution of this toxin to the main symptoms of envenoming process remains far unknown until now. Herein, we concomitantly performed *in vitro* and *in vivo* assays to show for the first time the dose-dependent response of crotamine-triggered hind limbs paralysis syndrome, up to now believed to be observable only at high (sub-lethal) concentrations of crotamine. In addition, *ex vivo* assay performed with isolated skeletal muscles allowed us to suggest here that compounds active on voltage-sensitive sodium and/or potassium ion channels could both affect the positive inotropic effect elicited by crotamine in isolated diaphragm, besides also affecting the hind limbs paralysis syndrome imposed by crotamine *in vivo*. By identifying the potential molecular targets of this toxin, our data may contribute to open new roads for translational studies aiming to improve the snakebite envenoming treatment in human. Interestingly, we also demonstrate that the intraplantal or intraperitoneal (*ip*) injections of crotamine in mice do not promote pain. Therefore, this work may also suggest the profitable utility of non-toxic analogs of crotamine as a potential tool for targeting voltage-gated ion channels in skeletal muscles, aiming its potential use in the therapy of neuromuscular dysfunctions and envenoming therapy.

## Author summary

Representing more than 10% of the dry weight of the crude venom of crotamine-positive rattlesnakes, crotamine may act as toxin mainly by imposing the physical immobilization of preys. Its presence was described to be important for antivenom therapy, although the knowledge on the effective contribution of crotamine to the main symptoms of envenoming process remains elusive and limited. Herein, we show for the first time the dose-dependent response for the hind limbs paralysis syndrome promoted by crotamine. We also report herein that compounds active on voltage-sensitive sodium and/or potassium ion channels can affect the positive inotropic effect elicited by crotamine *in vitro* in isolated diaphragm and also in the hind limbs paralysis syndrome triggered by crotamine *in vivo*. This potential targeting of voltage-sensitive sodium and/or potassium ion channels suggested here for crotamine may contribute to open new roads for translational studies aiming to improve the snakebite envenoming treatment in human. More importantly, nociceptive threshold evaluation demonstrated that crotamine does not trigger pain, and therefore, we also suggest crotamine as a potential tool for targeting voltage-gated ion channels present in skeletal muscles, with potential to be used as a lead compound to develop drugs for neuromuscular dysfunctions therapy.

## Introduction

Ophidian accident by *Crotalus* genus is mainly characterized by the myasthenic face with eventual paralysis of ocular muscles, and few reports of respiratory difficulties. Due to the characteristic myotoxic effects in envenoming by rattlesnakes, increased concentration of myoglobin in blood (due to rabdomyolysis) and increased serum enzyme as creatinine kinase (CK) and aspartate amino transferase (AST) is reported [1,2]. The main known components of South American *Crotalus* genus venom are the neurotoxin phospholipase A2 (namely crotoxin), the thrombin-like serinoprotease (namely gyroxin), a very potent aggregating protein (namely convulxin), and a small myotoxic polypeptide (namely crotamine). Therefore, *Crotalus* venom is relatively less complex compared to other snake genus venoms as those from *Bothrops* [1]. Most of the described myotoxic effects is believed to be determined by the phospholipase A2 crotoxin (the most abundant toxin), while the contribution of crotamine (the second more abundant toxin in the venom of crotamine-positive rattlesnakes) and other toxins present in the *Crotalus* venom seems to be marginal to the main symptoms of envenoming, and therefore, still remains little explored [1]. Interestingly, not all *Crotalus* snakes express crotamine in venom, which supported the subclassification of *Crotalus durissus terrificus* species as crotamine-positive and -negative specimens [3]. The presence of crotamine in the venom is considered of high importance for antivenom vaccine production [1], and consequently, also for pharmacotherapeutic interventions.

Crotamine is a polypeptide composed by 42 amino acid residues [YKQCHKKGGHCFPKEKICLPPSSDFGKMDCRWRWKCCKKGSG], which gives a molecular weight of about 5 kDa [4]. Structural similarity of crotamine with other snake myotoxins led to the suggestion of skeletal muscles as main target for the toxic effect of crotamine. Interestingly, the most characteristic effect observed after the injection of the purified native crotamine in preys (as rats and/or mice) is the hind limbs paralysis syndrome, which was observed only experimentally, after administration of high doses of pure crotamine, and which was also frequently followed by animal death, most probably due to respiratory failure [5]. In fact, the hind limbs paralysis was typically observed up to now at concentrations as high as 2.0 mg/kg of body weight (BW) for intraperitoneal (*ip*) injections in mice [6], while the LD_50_ for crotamine was described to be of about 0.07 to 6 mg/kg of BW for *ip* [7], 1.5–3.0 mg/kg BW for intravenous (*iv*) and 0.46 mg/kg BW for subcutaneous (*sc*) administrations [8,9]. Despite the fact that the skeletal muscles comprise about half of an animal body weight (BW), only ~1.5% of the total amount of native ^125^I-labeled crotamine injected by *ip* route reach this tissue in mice [6]. Even though, the contracture of isolated skeletal muscles triggered by crotamine in *ex vivo* assays was also described to occur only at high concentrations (*e*.*g*. above 10–50 μg/mL, which correspond to about 2–10 μM) [10]. Myoclonia, which is characterized by the involuntary twitching of a muscle or of a group of muscles in great muscle masses, was also reported in experimental crotamine-positive crotalic envenoming in cattle, but with no report of hind limbs paralysis [11].

The possible involvement of sodium (Na^+^) channel in hind limbs paralysis triggered by crotamine was initially hypothesized due to the observed inhibition of the crotamine-induce contracture of mice isolated skeletal muscle in the presence of Na^+^ voltage-gated channel blocker tetrodotoxin (TTX) [12]. However, under such specific *ex vivo* experiment condition, even a skeletal muscle contraction induced by direct electrical stimuli would be impaired under described TTX-induced blockage of Na^+^ channels [12]. In other words, as the TTX inhibition of the Na^+^ ions passage through these channels may prevent the nervous system from carrying the stimuli signaling, this blockage might necessarily hamper the muscle contraction in response to any kind of nervous stimuli, therefore, completely immobilizing the muscles and preventing the muscle response even for direct or indirect nerve evoked stimulations in *ex vivo* assays [12,13,14]. The same limitation would be also expected for the membrane labilizer veratridine (VTD), as the persistent activation of Na^+^ channels imposed by VTD determines a fatiguing stimulation, ultimately leading to an increased nerve excitability and consequent hyperpolarization [15].

Therefore, the reported involvement of ion channels in the hind limbs paralysis seems controversial up to now, with a single old report with feeble evidence suggesting the involvement of Na^+^ channels [12], although highly repeated by others with no further additive evidence supporting this hypothesis, and another more recently published work, using electrophysiological and isolated skeletal muscle contraction assays, ruling out the involvement of these same Na^+^ ion channels [16].

In fact, fast twitch skeletal muscles found in the hind limbs of rodents show higher presence of Na^+^ channels compared to slow-twitch muscles, as the soleus [17,18]. Higher susceptibility of fast contraction extensor digitorum longus (EDL) muscle to the action of crotamine compared to the slow contraction soleus muscle of mice was previously demonstrated [16]. However, the direct action of crotamine on several α-subunits of human Na^+^ channels (Na_v1.1_-Na_v1.6_) expressed in HEK293, as well in acutely dissociated dorsal root ganglion (DRG) neurons of mice, could not be confirmed in patch-clamp experiments [16].

Interestingly, other authors independently suggested the potential of crotamine to act on potassium (K^+^) ion channels [19,20]. As toxins that block voltage-gated K^+^ channels and also those that modify Na^+^ channels gating equally exhibit positive inotropic effects on isolated skeletal muscles [21,22,23], another possible target for the action of crotamine could be the K^+^ channels. Yount et al. [19] were the first to suggest the crotamine action on K^+^ channels which was later confirmed by others conducting electrophysiological studies with voltage-dependent Kv_1.1_, Kv_1.2_ and Kv_1.3_ channels [20]. Nevertheless, to the best of our knowledge, the involvement of the K^+^ channels in the hind limb paralysis syndrome triggered by crotamine was never evaluated up to now.

Therefore, currently, the molecular mechanism(s) underlying the hind limbs paralysis syndrome triggered by crotamine persists poorly understood, and the potential molecular target(s) for crotamine in skeletal muscle still remain(s) unidentified. Herein, the effects of different concentrations/doses of crotamine were evaluated in both *ex vivo* (isolated skeletal muscle under direct electrical stimulation) and *in vivo* (observation of hind limb paralysis in living mice) assays, aiming to evaluate the influence of active compounds on Na^+^ and/or K^+^ channels in the animal death and/or skeletal muscles contraction elicited by crotamine. By employing the open field and sucrose splash tests, we also investigated the potential change(s) in animal behavioral due to the *ip* administration of crotamine. In addition, abdominal writhing, electronic von Frey and tail-flick tests were performed to preclude any possible influence of crotamine-induced hypernociception in these behavioral assays.

## Materials and methods

### Drugs and solutions

Crude venom of *Crotalus durissus terrificus* rattlesnakes was obtained from the snake colony housed in the serpentarium of the Faculdade de Medicina de Ribeirão Preto, São Paulo University—Ribeirão Preto (USP-RP). Crotamine was kindly prepared and purified by Dr. Eduardo B. Oliveira (authorization of access to genetic resources No. 010426/2010 COAPG/DABS/CNPq, term of concession No. 20100104268), essentially following the procedure described by Hayashi et al. [24]. Salts and all other chemicals used in this study were of the best quality available (*i*.*e*., ACS grade), and they were all purchased from Sigma Aldrich (St. Louis, Missouri, USA).

### Animals

All animals were from the Laboratory of Animal Experimentation (LAE) of the Institute of Pharmacology and Molecular Biology (INFAR) of Universidade Federal de São Paulo (UNIFESP). They were kept under controlled conditions of temperature and illumination (12 h light/dark cycle), in a temperature-controlled environment (22 ± 2°C), with free access to water and food. The Research Ethics Committee of UNIFESP approved all protocols performed in this study (CEUA No. 7948150915), and all procedures were conducted in accordance with the U.K. Animals (Scientific Procedures) Act, 1986 and associated guidelines, EU Directive 2010/63/EU for animal experiments, or the National Institutes of Health guide for the care and use of Laboratory animals (NIH Publications No. 8023, revised 1978).

### *Ex vivo* assays

**Fig 1:**
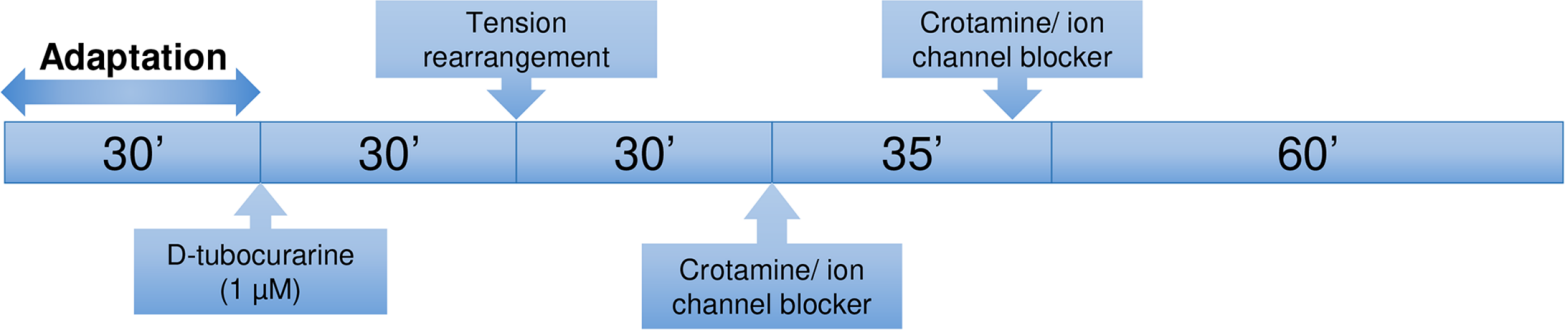
Schematic representation of the *ex vivo* assays.

For isolated diaphragm skeletal muscle preparation, male Swiss mice of 90 to 120 days old were euthanized by cervical dislocation, and the hemi-diaphragms were dissected before the washing with Tyrode nutrient solution (135 mM NaCl, 5 mM KCl, 1 mM MgCl_2_.6 H_2_O, 15 mM NaHCO_3_, 2 mM NaH_2_PO_4_ H_2_O, 2 mM CaCl_2_.2 H_2_O, 11 mM D-glucose in distilled water, pH 7.4), and each hemi-diaphragm was mounted as previously described by Duarte et al. [25]. Briefly, each hemi-diaphragm segment was transferred to an organ bath filled with 5 mL of Tyrode’s solution at 37ºC, and the dissected tissue was tied up to a holder fixed at the bottom of the glass organ bath, while the upper extremity of the tissue was fixed to a force transducer, adjusted to impose an optimal tension (*i*.*e*. a condition in which the maximum contraction of each skeletal muscle preparation is observed). The contraction of the diaphragm muscle was induced by transmural electrical stimuli conducted through the platinum electrodes, at a frequency of 0.1 Hz and under supramaximal voltage, with duration of 2 ms each. After 30 min of stabilization of the skeletal muscle contraction amplitudes, the nicotinic acetylcholine receptor antagonist *d*-tubocurarine (1 μM) was added to the preparations to avoid the interference of acetylcholine released from the presynaptic terminals. After 30 min of stabilization of contraction amplitudes in the presence of *d*-tubocurarine, the tension of the preparation was rearranged. The influence of crotamine on the isometric twitch contraction was evaluated for additional 1.5 h, after confirming the stabilization of contraction amplitude, in the presence or absence of K^+^ ion channel blockers [26], which were added to the preparation at least one hour before the end of the experiment. Experiments with previous addition of the K^+^ ion channel blockers were conducted exactly in the same way, and crotamine was applied 35 min after the addition of the channel blocker as schematically demonstrated (Fig 1).

Contractions of the skeletal muscle preparations were recorded using the Power Lab force transducer and the obtained data were analyzed using the Power Lab Chart 6 software (AD Instruments, Australia). Twitch amplitudes (mean ± SE) were expressed as percentage of the basal values (100%), as observed immediately before the addition of crotamine or ion channel blockers into the preparation.

### *In vivo* assays

Male C57/BL6 mice of 90 to 120 days old (25–30 g body weight, BW) were used for the *in vivo* experiments.

#### Open field

**Fig 2:**
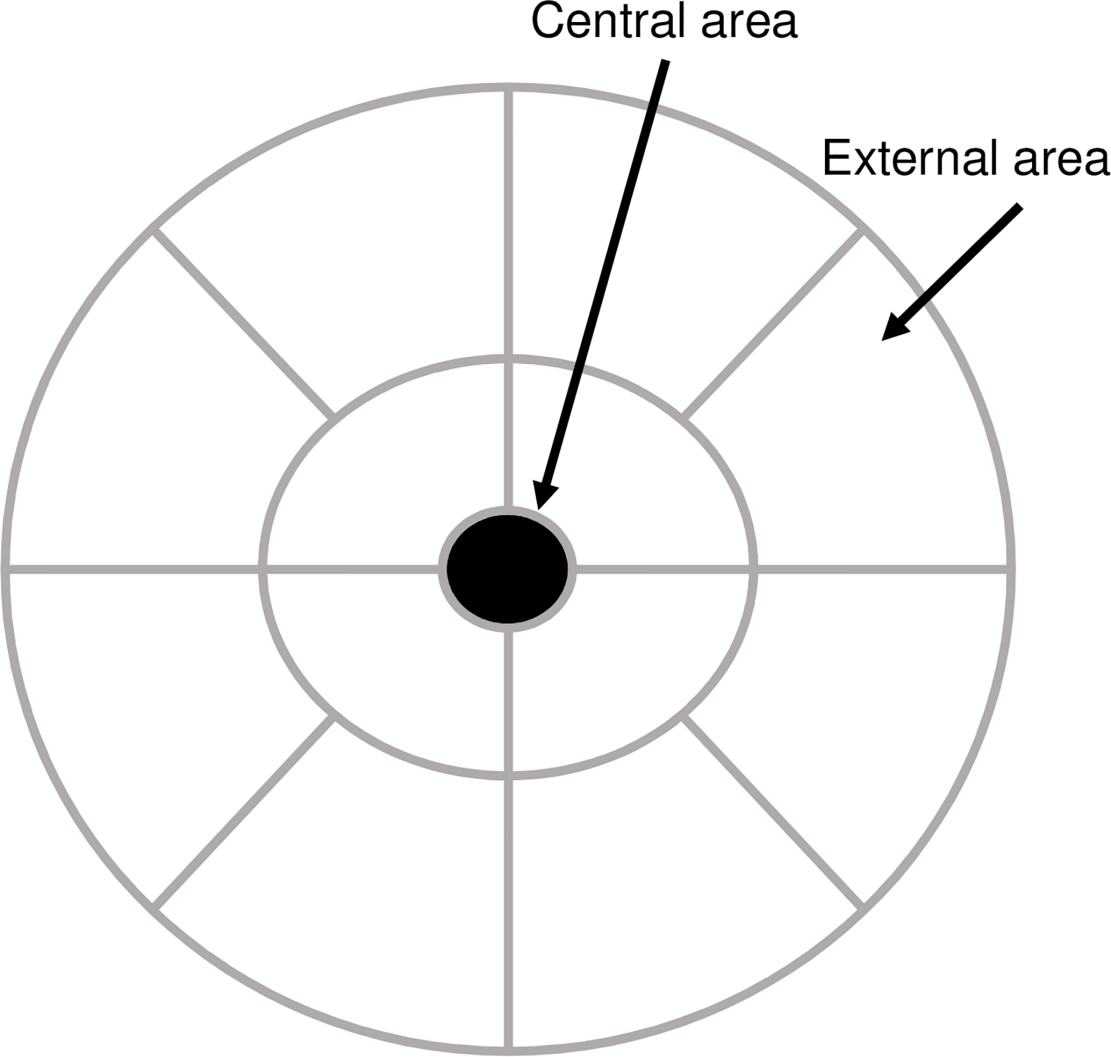
Schematic representation of the areas of the open field arena floor.

The experiments were performed in an open field arena (40 cm in diameter surrounded by walls 50 cm high, and white floor designed with black lines as presented in the scheme below), in which animals were placed on the center, after the intraperitoneal (*ip*) administration of different doses of crotamine (7.5, 15 and 30 μg/animal, which correspond to approximately 0.3, 0.6 and 1.2 mg/kg BW), or saline (used as control), in a final volume of 100 μL/animal. The animals were placed in the open field and the sessions were recorded, using a digital camera placed above the apparatus, for a period of 1 h after the *ip* injection of crotamine or voltage-activated K^+^ channel blocker 4-aminopyridine (4-AP, 30 μg/animal, which corresponds to 1.2 mg/kg BW), or of vehicle saline (negative control) alone. For the treatments with crotamine and ion channels blockers together, each one was injected 10 min before the other compound administration. Behavioral parameters were collected and analyzed from the video-recorded sessions employing an animal tracking software (Anymaze, Stoelting, USA). The distance traveled by the animals in the apparatus (total traveled distance in each session (in meters)), number (N) of entries into the central or external areas (Fig 2), and the amount (N) of stools left in the apparatus after each session were registered. In addition, the hind limbs paralysis of the animal due to crotamine administration was also monitored by visual observation after tactile stimulation. For last, the time lapse, after the injection of crotamine, in which the paralysis occurred was recorded (in min).

#### Sucrose splash

**Fig 3:**
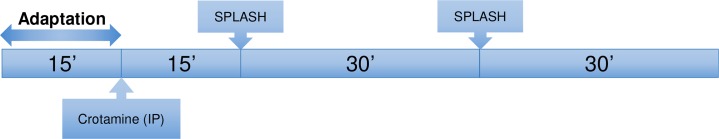
Schematic representation of the sucrose splash test.

The sucrose splash tests were performed as schematically demonstrated (Fig 3). Animals were placed in the experimental box (17.6 × 28.7 × 12.5 cm) with sawdust bedding, for adaptation of 15 min (one animal at a time), and then each animal received a single *ip* administration of 7.5 μg of crotamine/animal (approximately 0.3 mg/kg BW, in a final volume of 100 μL/animal), or saline in control animals. Then, the first splash of 5% sucrose solution was applied 15 min after crotamine or saline administration by *ip* route. A second splash was applied 30 min after the first splash, and the animals were continuously monitored along the complete experimental period. The entire experiment was video recorded, and the duration of grooming behavior(s) (in a time window of 5 min each) was measured by watching the video at the end of experiments. The total sum of grooming period was also considered for the analysis.

### Pain threshold evaluation

#### Assessment of animal abdominal writhing

Immediately after administration of crotamine (*ip*, 15 μg/animal, which corresponds to 0.6 mg/kg BW, in a volume of 100 μL), mice from the experimental group (n = 4) and control group receiving saline (n = 4) were observed by naked eyes for abdominal constriction in the form of muscle contraction and/or hind limbs elongation [27]. The nociception intensity was quantified by counting the total number of writhes within 30 min after the injection of crotamine.

#### Mechanical hypernociception evaluation by electronic von Frey test

The mechanical nociceptive threshold was assessed by the electronic von Frey test (Insight Equipamentos Ltda., Ribeirão Preto, SP, Brazil). The test was applied before (basal measure), and after (15 and 30 min) the intraplantar injection of crotamine (1 or 5 μg/animal paw) or saline, following a previously described protocol [28]. Briefly, the animals were placed suspended in acrylic boxes, approximately 30 cm from the workbench, with a wire bottom to permit the access to the paws during the test. Animals were kept in the boxes for 30 min before the basal measurement, to allow the acclimation. A hand-held force transducer connected to a digital counter, which automatically register the force (in g), was applied on the paw. The instrument was calibrated to register a maximum force of 150 g, maintaining the accuracy of 0.1 g until 80 g of pressure force. The maximum force applied in mice paws was of about 18 g. The contact of the transducer with the paw occurred through a polypropylene disposable tip of 0.5 mm^2^. A perpendicular crescent force was applied to the central area of the hind limb of the animals, which was automatically stopped when the animals presented the reaction of withdrawing the paw. The stimulation was repeated until the animals presented two similar measures, and all testing was blind regarding to the group designation, *i*.*e*., experimental (crotamine-treated) or control group (receiving saline).

#### Tail-flick test

A radiant heat analgesiometer (Tail Flick Analgesia Meter, Columbus Instruments, Columbus, OH, USA) was used as described [29,30]. Each mouse was tested immediately before and 30 min after the injection of crotamine (*ip*, 15 μg/animal, which corresponds to 0.6 mg/kg BW) or saline (control group). Radiant heat from a halogen lamp (8 V, 50 W) was focused on the lower third of the tail of animals placed in a restraint cage with their tails protruded from the apparatus. The light intensity was adjusted to achieve the baseline latencies of 2–3 sec, and the cut-off was determined as 8 sec, to avoid damage of tail or unnecessary harm to the animal.

#### Hind limb paralysis and animal death evaluation

Evaluation of the hind limbs paralysis due to the acute *ip* administration of crotamine was conducted after injection of the Na^+^ channel blocker tetrodotoxin (TTX, 5 μg/animal, *ip*, which corresponds to 166 mg/kg BW), which dose was the same as previously described [31,14]. TTX was injected 10 min prior to the administration of crotamine (100 μg/animal, which corresponds to approximately 4 mg/kg BW) or of veratridine (VTD), which is a ligand of activated Na^+^ channels that abolishes the inactivation of Na^+^ channels [32]. For evaluating the influences of VTD on crotamine effects *in vivo*, VTD (0.5 to 6.25 mg/kg BW) was injected 10 min before or after the administration of crotamine (30 μg/animal, which corresponds to 1.2 mg/kg BW). Similarly, for the experiments with the inhibitors of voltage-activated K^+^ channels, namely 4-AP (30 μg/animal, which corresponds to 1.2 mg/kg BW) [31], crotamine (30 μg/animal, which corresponds to 1.2 mg/kg BW) was injected 10 min before or after the administration of each K^+^ channel blocker. In all experiments, saline was used as negative control. The injection volume for all conditions was of 100 μL/animal. The animals were monitored for a period of 90 min after the administration of crotamine or other drugs, and the hind limbs paralysis and eventual animal death were both assessed by visual observation (naked eyes) after tactile stimulation.

#### Statistical analysis

Two-way ANOVA analysis was used to identify differences in muscle force and frequency among the different experimental conditions. One-way ANOVA analysis was applied to investigate effects of treatment on total distance travelled, center and external explorations and amount of stool in the open field. Repeated ANOVA measures were applied to investigate the time versus treatment effects on travelled distance (in meters) in the open-field and grooming behavior period (in second) in defined time windows along the sessions. Tukey’s post-hoc was performed to point out the specific differences following ANOVA analysis. Unpaired two-tailed t-test was used to compare total grooming observed in each group during the splash test. Statistical analyses were performed in Prism version 6.0 (GraphPad Software, San Diego, CA). Values of *p* < 0.05 were considered to indicate the statistically significant differences for comparison between the experimental and control groups.

## Results

### *Ex vivo* assays

**Fig 4:**
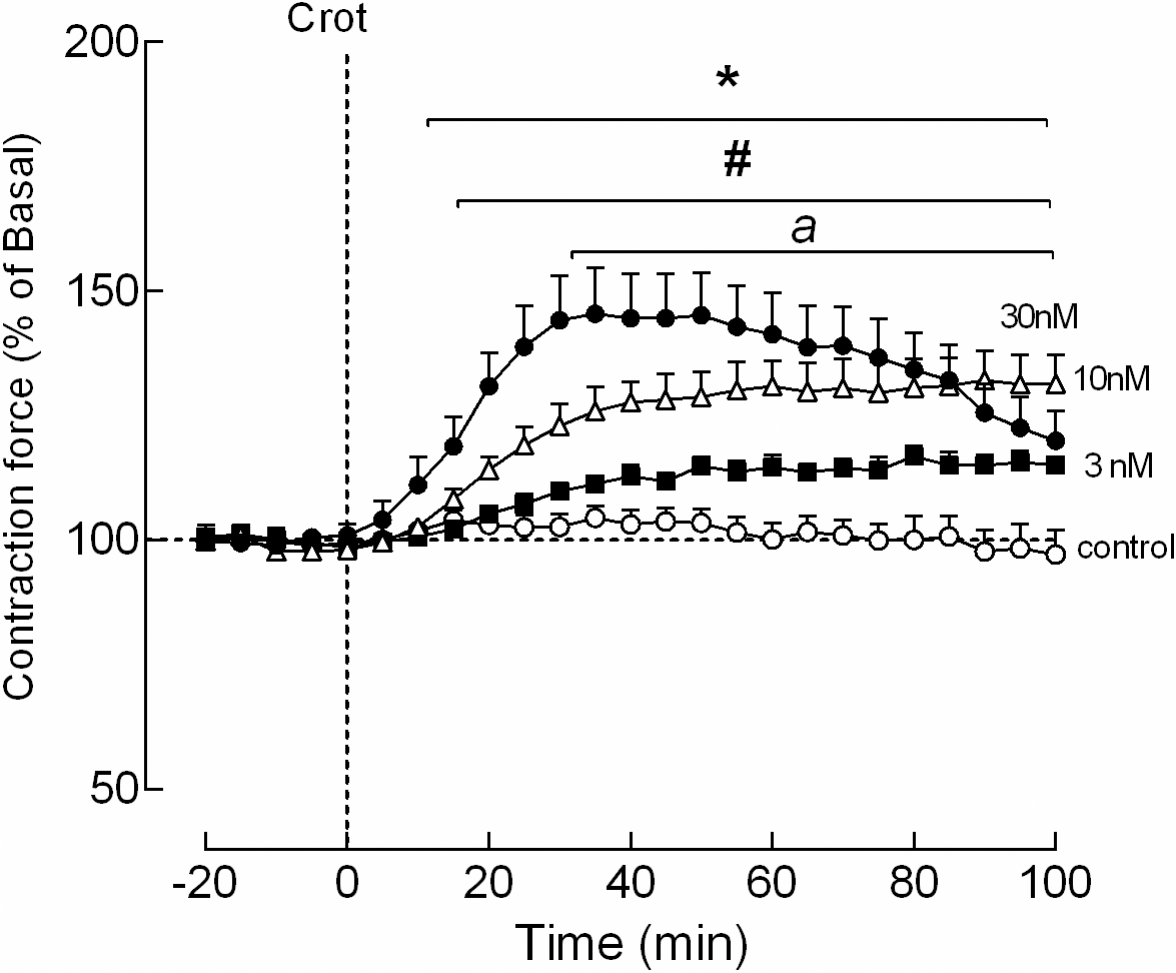
Effect of crotamine on contraction force of isolated diaphragm of mice. The contraction of isolated mouse diaphragms was induced by transmural electrical stimulation (0.1 Hz, 2 ms duration, under optimum voltage and supramaximal voltage) and the isometric contractions were determined by plotting the average value of contraction force of each 5 min. Crotamine (from 3 to 30 nM) increased by about 17 to 46% the amplitude of twitch-contraction of isolated muscle induced by the direct transmural electrical stimuli. The time of crotamine addition was considered as zero (vertical dashed line), and the baseline contraction amplitude was considered as 100% (horizontal dashed line). The results (in percentage) were expressed as mean ± SEM (N = 6 independent experiments). The value *p* < 0.05 for ANOVA statistical analysis compared to the control was considered significant as indicated by *, ^#^ and ^*a*^, for 30, 10 and 3 nM of crotamine, respectively.

Several concentrations of crotamine (*i*.*e*., 3, 10, 30 and 100 nM, which correspond to about 0.015, 0.05, 0.15 and 0.5 μg/mL, respectively) were used here to evaluate the effects of crotamine on the contraction of isolated mouse diaphragm muscle, which was reported to present high expression of sodium ion (Na^+^) channels [13]. At 100 nM of crotamine (about 0.5 μg/mL), we observed an increase in the baseline resting tension, which is characteristic of muscle contracture due to muscle lesion. Therefore, only concentrations below 30 nM of crotamine were considered for the concentration-response analysis. At 30 nM, crotamine increased the muscle contraction force by 38.2 ± 5.0%, while at 10 and 3 nM, the increase in the contraction force were of about 32.1 ± 4.0% and 17.1 ± 1.4%, respectively (Fig 4).

**Fig 5:**
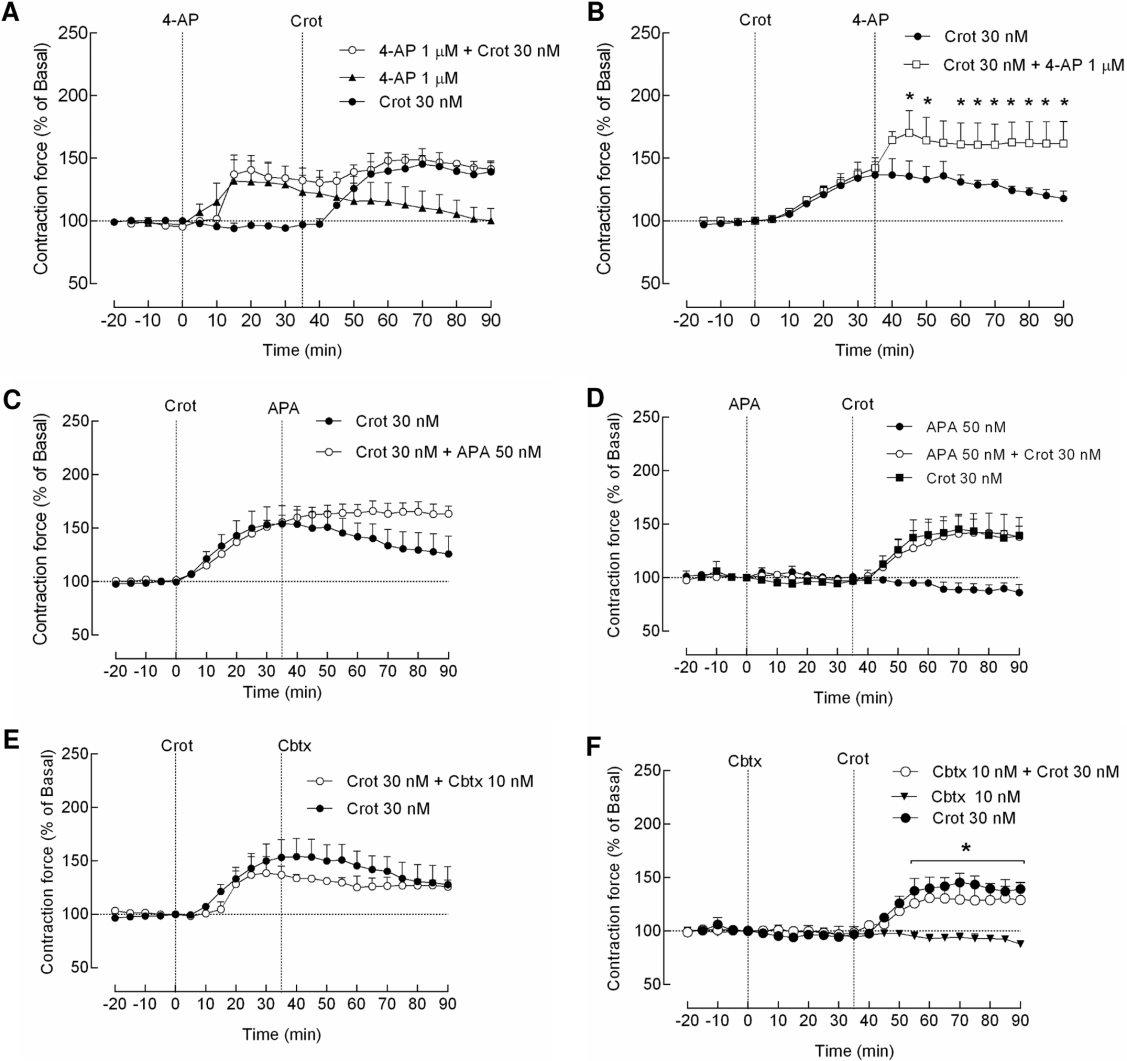
Effect of K^+^ channel blockers on the effect of crotamine on the contraction force of isolated diaphragm of mice. The increases of muscle contraction force induced by crotamine (30 nM) were evaluated in the presence of the voltage-dependent K^+^ channel blocker, namely 4-aminopyridine (4-AP, 1 μM) (panels A and B), and Ca^2+^-dependent K^+^ channel blockers, namely apamin (APA, 50 nM) (panels C and D) and charibdotoxin (CBTx, 10 nM) (panels E and F), which were added into the preparation after (panels A, C and E) or before (panels B, D and F) the addition of crotamine. Crotamine or channel blockers were applied at time zero or 35 min after the first compound addition, as indicated by the vertical dashed lines. The baseline contraction amplitude was considered as 100% (horizontal dashed line). The results (mean ± SEM, N = 6 independent experiments) were expressed as a percentage. *p < 0.05 for ANOVA statistical analysis compared to their respective control.

Considering the reports suggesting the potassium ion (K^+^) channels as potential target for crotamine [19,20], we also evaluated the effects of two classes of K^+^ channels blockers, namely apamin (APA) and 4-aminopyridine (4-AP), which are selective blockers of Ca^2+^-activated K^+^ channels and blockers of members of K_v_1 family of voltage-activated K^+^ channels, respectively [26], in the muscle contraction force increases elicited by crotamine. In the present experimental condition, the pre-incubation with the voltage-dependent K^+^ channels inhibitor 4-AP (1 μM) did not affect the positive inotropic effect of crotamine (30 nM) on skeletal muscle contraction force (Fig 5A), but a significant increase in the contraction force (~20%) was clearly observed soon after the addition of 4-AP, when added after a previous incubation for about 35 min with crotamine (Fig 5B). Interestingly, no influence on the contraction force increases induced by crotamine (30 nM) was observed after a pre-incubation with APA (50 nM) or with the Ca^2+^-activated K^+^ channels blocker charibdotoxin (CBTx, 10 nM) (Fig 5D and 5F, respectively). The posterior addition of APA showed only a non-significant trend for increasing the positive inotropic effect of crotamine on isolated skeletal muscle (Fig 5C), while no statistically significant differences were observed for the addition of CBTx after crotamine (Fig 5E).

### *In vivo* assays

#### Open field test

**Fig 6:**
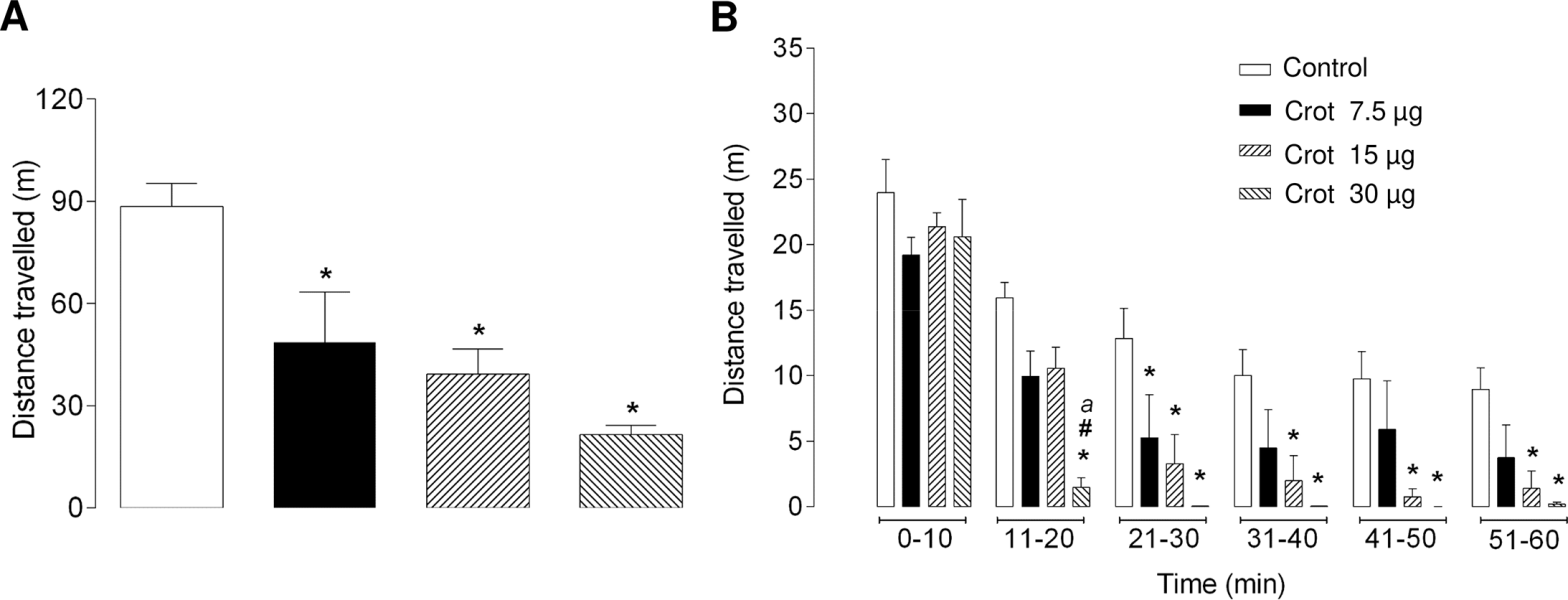
Mean distance traveled by the animals after the administration of different doses of crotamine. The dose-response effect of crotamine was confirmed by the differences in the mean distance traveled by the animals after receiving 30, 15 or 7.5 μg/animal (which correspond to 0.3, 0.6 and 1.2 mg/kg BW, respectively) by intraperitoneal (*ip*) injection, quantified during 60 min **(A)** or measured in periods of each 10 min **(B)**. Total locomotor activity was continuously monitored during 60 min, and each bar in the graphic represent the average traveled distance (in meters) of at least 6–7 animals/group. The value of *p* < 0.05 for ANOVA statistical analysis was considered significant. *, ^#^ and ^*a*^ represent the comparison with control, 7.5 μg and 15 μg of crotamine per animal, respectively.

**Fig 7:**
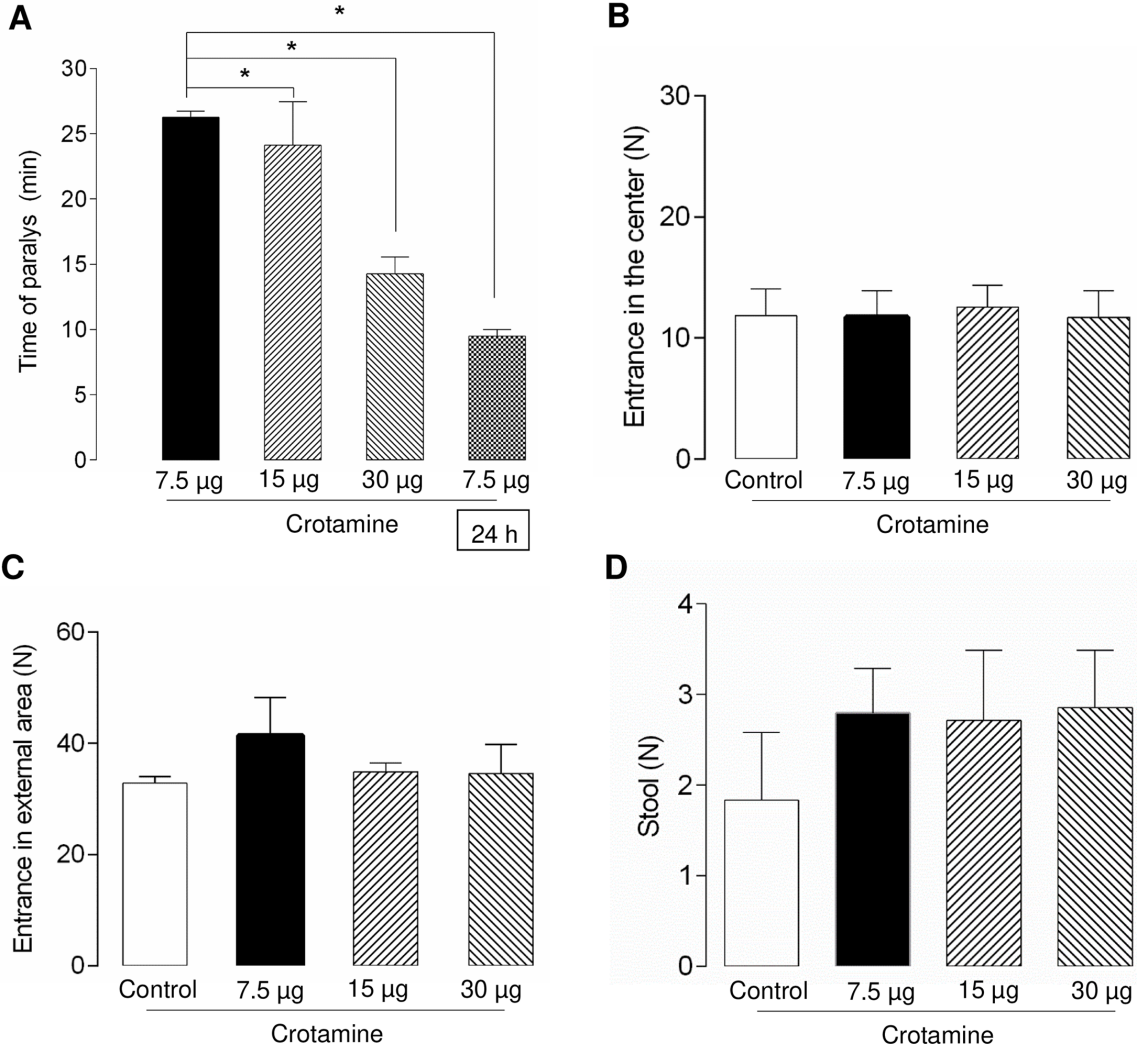
Evaluation of paralysis time and animal behavior in the open field. Time of paralysis (**A**), number of times that the animal crossed the central area of the open field (**B**) and the external area of the open field (**C**), and amount of stools deposited by the animal in the field (**D**) were recorded and expressed in minutes (min) for time of paralysis onset (in A) and in numbers of events (N) for B, C and D. Different doses of crotamine (7.5, 15 or 30 μg/animal which correspond to 0.3, 0.6 and 1.2 mg/kg BW, respectively) was administered by intraperitoneal (*ip*) injection. The last bar in panel A, indicated by the 24 h in the open box, refers to the *ip* administration of same dose of crotamine (7.5 μg/animal or 0.3 mg/kg BW) in the same animal 24 h after first crotamine administration, showing a significant decrease in the time for paralysis onset compared to the first administration of crotamine in naïve animal, a day before. *p < 0.05 for ANOVA statistical analysis.

The open field test was used to observe the eventual time-response or gradual decrease of the skeletal muscle functioning. Animals receiving a single *ip* injection of different doses of crotamine (7.5, 15 and 30 μg/animal which correspond to 0.3, 0.6 and 1.2 mg/kg BW, respectively) showed significant decreases in the total distance travelled in a period of 1 h [F(5.138) = 51.03; *p* < 0.0001], which were proportional to the dose of crotamine administered. The decreases in the total distance travelled imposed by all doses of crotamine evaluated here were also statistically different compared to the control group. For instance, control animals travelled for about two times the distance travelled by the animals receiving the lowest dose of crotamine (7.5 μg/animal or 0.3 mg/kg BW) (Fig 6A). The data plotted for timeframes of 10 min showed that, for the highest dose of crotamine (30 μg/animal or 1.2 mg/kg BW) evaluated, the first statistically decrease of the ambulatorial displacement was observable as early as 10 min after the *ip* injection of crotamine (Fig 6B) (*i*.*e*., at the 11–20 min timeframe the hind limb paralysis was settled, Fig 7A). On the other hand, at lower doses (*e*.*g*. 7.5 or 15 μg/animal), crotamine decreased the travelled distance only after longer periods, and statistically significant decrease in ambulatorial displacement was noticeable only after 20 min of monitoring (Fig 6B). In summary, the average time for paralysis onset was of about 26.3 ± 1.0, 24.1 ± 8.7 or 14.3 ± 3.4 min after the *ip* injection of 7.5, 15 and 30 μg of crotamine/animal, respectively (Fig 7A). In other words, all doses of crotamine showed significant decrease in locomotion of crotamine-treated compared to the control group animals, in which the time of paralysis was inversely proportional to the administered doses of crotamine.

Interestingly, repeated *ip* injection of same dose of crotamine (7.5 μg/animal or 0.3 mg/kg BW) in the same animal, 24 h after, showed significant improved effect of the toxin on paralysis, as the time for the hind limb paralysis onset was decreased by about 38.2% compared to that determined for the administration of the same crotamine dose in naïve animal (Fig 7A, column indicated as 24 h). The frequency of crossing the external area of the open field, the frequency of animal crossing the central area of the open field, and the amount (number, N) of stools deposited by the animals in the open field during the experimental period were also recorded, although no significant differences were observed for any of these parameters (Fig 7B, 7C and 7D), as confirmed by the statistical analysis by one-way ANOVA followed by Turkey’s test.

#### Evaluating the participation of K^+^ channels in the hind limbs paralysis in vivo

**Fig 8:**
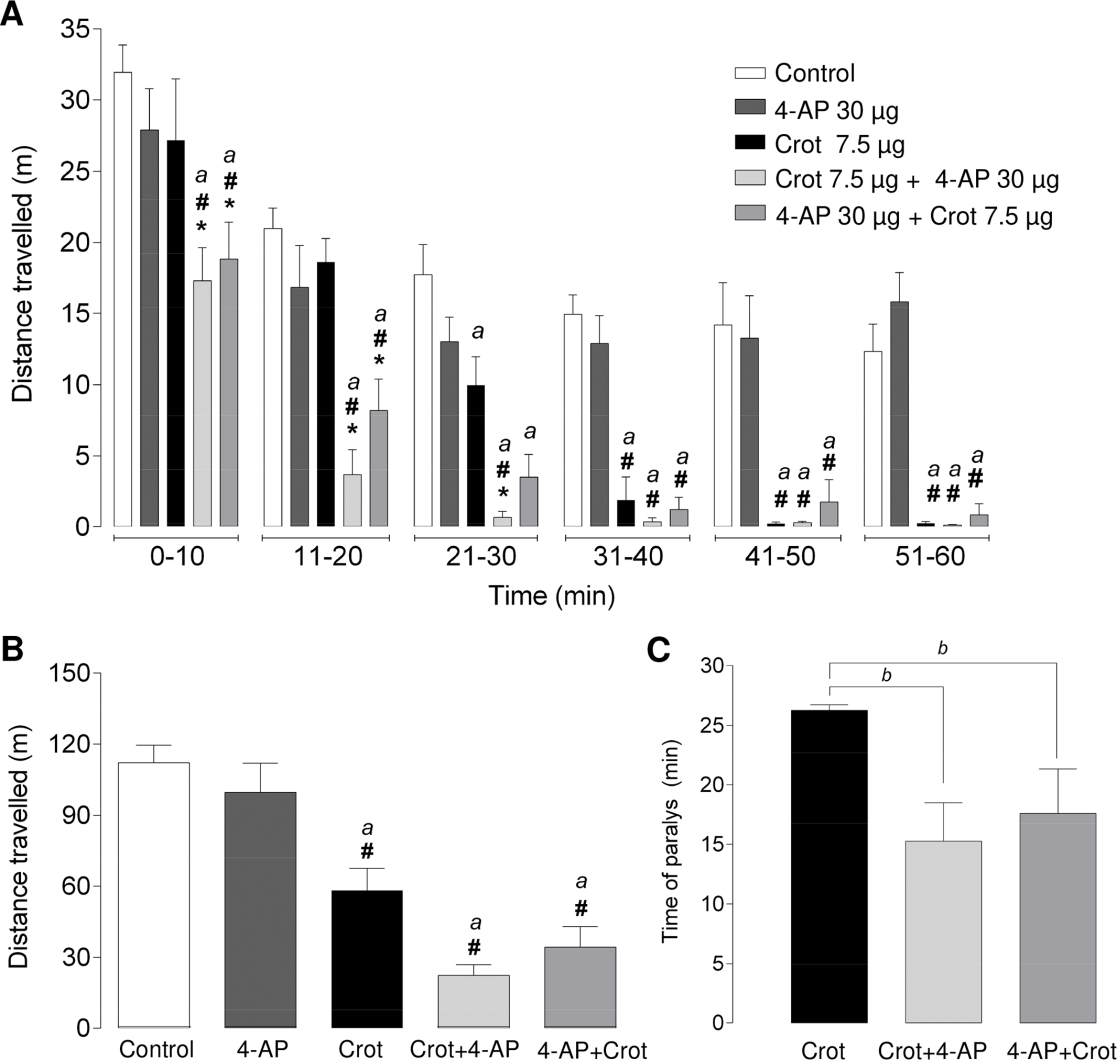
Mean distance traveled by animals after the administration of crotamine and the voltage-dependent K^+^ channels blocker 4-AP. The distance travelled by the animal treated with crotamine and 4-AP was quantified in timeframe periods of 10 min each (**A**), as well as the total distance travelled during 60 min was detemined (**B**), and the time of paralysis was also recorded (**C**). The values were considered significant for *p* < 0.05 for ANOVA statistical analysis. *, ^#^ and ^*a*^ in panels A and B refer to comparisons with control, 4-AP (30 μg/animal, which corresponds to 1.2 mg/kg BW) and crotamine (7.5 μg/animal or 0.3 mg/kg BW), respectively, while ^*b*^ refers to comparisons with animals receiving only crotamine.

The administration of the inhibitor of voltage-activated K^+^ channels belonging to the Kv1 family, namely 4-AP (30 μg/animal, which corresponds to 1.2 mg/kg BW), significantly potentiated the hind limbs paralysis elicited by crotamine, as denoted by the significant decrease of total distance travelled by the crotamine-treated animals (Fig 8A and 8B). This prominent decrease in ambulatorial displacement could be observed not only when the travelled distance was evaluated for the total experimental period, which was of 1.5 h after the *ip* administration of the toxin (Fig 8B). However, when the ambulatorial displacement was analyzed in timeframe periods of 10 min, a significant decrease in the animal travelled distance was first noticed only 20 min after the administration of crotamine alone, while after administration of crotamine with 4-AP, it was observed before the 20 min (Fig 8A). In other words, the crotamine ability to paralyze the skeletal muscles was potentiated by the administration of 4-AP before or after crotamine injection, as denoted by the earlier hind limbs paralysis onset compared to that observed for crotamine administered alone (Fig 8C).

#### Sucrose splash test

**Fig 9:**
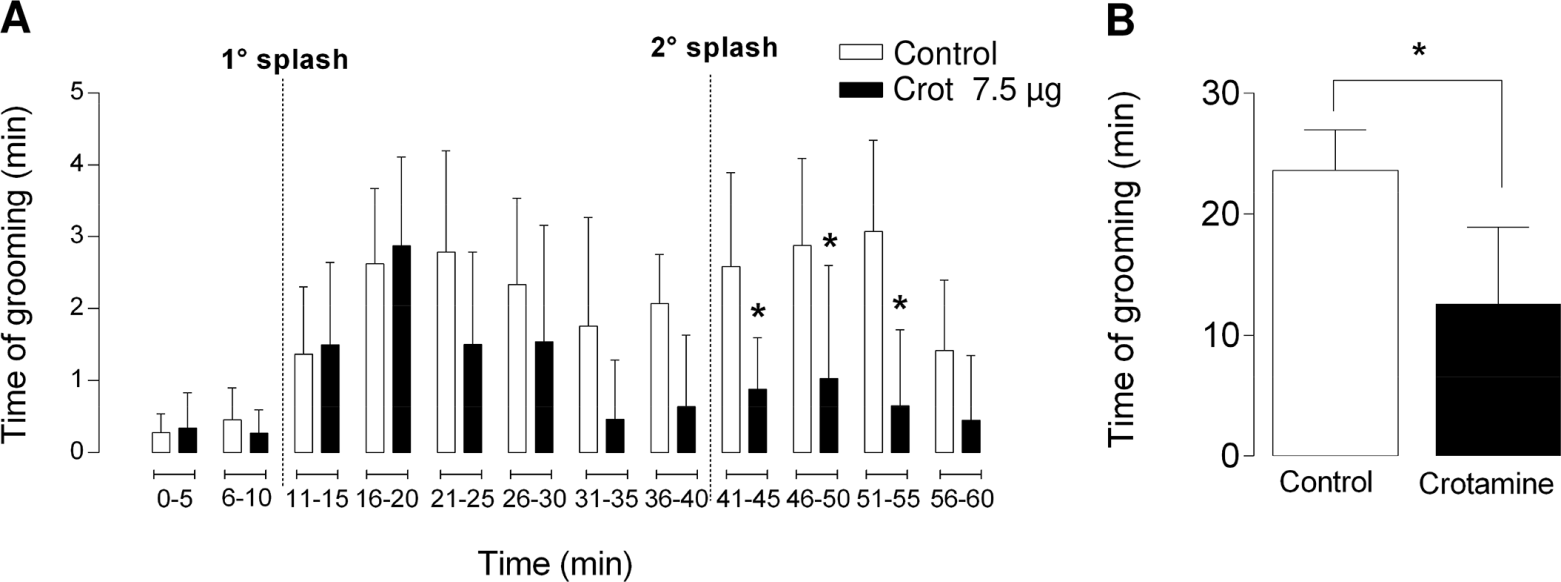
Sucrose splash test. The period of grooming denotes the time spent by the animal in self cleaning activity, and the grooming was quantified for periods of 5 min each, statistically significant differences between animals receiving crotamine (7.5 μg/animal or 0.3 mg/kg BW) by intraperitoneal (*ip*) route compared to vehicle receiving control animals were observed only after the second splash (A). The animals (N = 9) were continuously monitored this experiment during 1.5 h, and two splashes were performed in this interval, as indicated by the vertical dashed lines. The significant decreased activity was observed in the animals that received crotamine (average 754 s) compared to control animals (average 1498 s) was observed (B). The results (mean ± SE) were expressed as grooming period (s). * *p* ≤ 0.05 for unpaired two-tailed t-test was used to compare total grooming observed in each group during the splash test.

The sucrose splash test consists of evaluating the mouse grooming behavior, which requires the use of the animal front paws [33,34]. Therefore, this test was conducted here aiming to verify if the effects of crotamine were limited to the hind limbs or if the front paws movements were also affected by this toxin. The time spent for the cleaning behavior (grooming) observed along the entire experiment, which lasted for up to 1.5 h with two splashes in the period, was computed. The average period of time for grooming of animals receiving crotamine (7.5 μg/animal or 0.3 mg/kg BW) by *ip* injection was of about 754.5 ± 126.9 sec, while for the control animals was of about 1418.0 ± 67.1 sec (Fig 9). Although the observed significant decrease of the total grooming behavior of mice receiving single dose of crotamine (7.5 μg/animal, which corresponds to 0.3 mg/kg BW), we supposed this feature was not determined by the paralysis of the front paws, as the animal was still able to move these front paws in response to the tactile stimulation or by suspension of the animal tail and hind limbs (data in S1 Video).

#### Pain threshold evaluation

**Fig 10:**
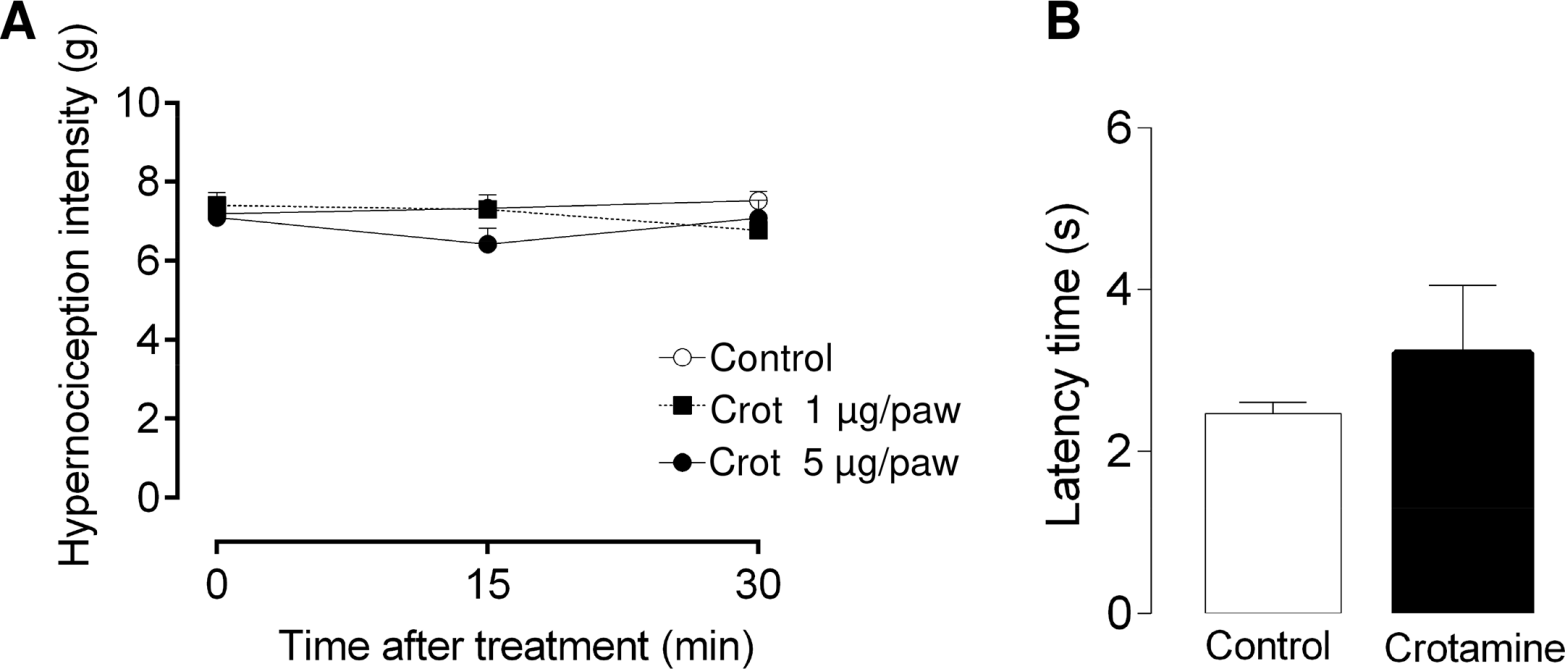
Pain threshold evaluation. The administration of crotamine (1 or 5 μg/animal paw), by intraplantar injection, did not show significant difference in hypernociception intensity (in grams (g)) as evaluated by Electronic von Frey test (A). The Tail-Flick Analgesia Test was also used to test the animals 30 min after the injection of crotamine (*ip*, 15 μg/animal, which corresponds to 0.6 mg/kg BW) showing no significant difference in the latency time (s) compared to the control animals receiving the vehicle (B). *p < 0.05 for ANOVA statistical analysis.

Aiming to rule out that the main effects observed for crotamine in the open field or in the grooming and cleaning behaviors were not due to eventual pain determined by this toxin injection, the nociceptive threshold was additionally assessed by observing the abdominal writhing (which is a chemical-induced pain model), and also by employing electronic von Frey (which is a mechanical-induced pain model) and tail flick (which is a thermal-induced pain model) tests. Mice receiving crotamine (*ip*, 15 μg/animal, which corresponds to 0.6 mg/kg BW) did not present noticeable abdominal contortion, characteristic of abdominal writhing, during the observed period (30 min). Hence, no significant difference between crotamine-treated and vehicle-treated control animals was observed for the electronic von Frey and tail flick tests, after intraplantal (1 or 5 μg/animal paw) and/or *ip* injection of crotamine (15 μg/animal, which corresponds to 0.6 mg/kg BW), respectively (Fig 10).

#### Hind limbs paralysis in vivo

Although Chang and Tseng [10] have suggested that tetrodotoxin (TTX, 1 μg/mL) is able to prevent the stimulatory effects of crotamine (2 μg/mL, which correspond to about 400 nM) on single twitch response of isolated diaphragm in *ex vivo* assays, it is important to consider the limitation of using such *ex vivo* preparations to evaluate the effects of activators or blockers of Na^+^ voltage-gated channels, as they can completely block the skeletal muscle contraction ability, potentially including those induced by direct electrical stimuli [11,12,13,14]. For this reason, in this work, we choose to evaluate the effect(s) of these ion channels blockers *in vivo*. Different doses of crotamine administered by *ip* injection in mice showed here an unfamiliar dose-response for the hind limb paralysis onset, and statistically significant decrease of the time elapsed for the paralysis onset was observed after injection of higher doses of this toxin (Figs 4 and 5, Table 1). Actually, the *ip* injection of 30 or 50 μg of crotamine per animal, which corresponds to about 1.2 or 2.0 mg/kg BW, led to observable paralysis about 7 or 3 min after administration of toxin, respectively, followed by animal death after about 40 min (Table 1). In addition, the prior *ip* injection of TTX (5 μg/animal which corresponds to about 166 mg/kg BW) did not avoid the hind limbs paralysis or the animal death trigged by a single lethal dose of crotamine (30 or 100 μg/animal which correspond to about 1.2 or 4 mg/kg BW, respectively) *ip* injected in mice. Under this specific condition, the posterior injection of TTX, paralysis of the hind limbs elicited by crotamine was promptly observed (*i*.*e*. about 6 min after the injection of 30 μg of crotamine/animal). In the same way, animal death was observed about 40 min after the injection of crotamine, even if TTX was previously administered, therefore with no significant change for animal death effect compared to crotamine administered alone (Table 1). However, it is important to remark that the previous injection of TTX significantly augmented the time elapse for the paralysis onset determined by the *ip* injection of crotamine (30 or 100 μg/animal which correspond to about 1.2 or 4 mg/kg BW) (Table 1).

**Table 1:** Paralysis of mice hind limbs after intraperitoneal route administration of crotamine and/or ion channel active compounds.

Drug	Dose (mg/kg)	Time of paralysis	Animal death (time of death)
**Crot**	1.2	7’	Yes (~40 min)
	2.0	3’	Yes (~40 min)
**VTD**	0.5	No	No
	1.5	3’	No
	6.25	2’	No
**Crot + VTD**	1.2 + 1.5	6’	Yes (> 24 h)
**VTD + Crot**	1.5 + 1.2	7’	Yes (~40 min)
**TTX**	166	No	Yes (~20 min)
**Crot + TTX**	1.2 + 166	6’	Yes (~40 min)
**TTX + Crot**	166 + 4	8’	Yes (~40 min)
**TTX + Crot**	166 + 1.2	17’	Yes (~40 min)
**TTX + VTD**	166 + 1.5	No	No
**VTD + TTX**	1.5 + 166	3’	No
**4-AP**	1.2	No	No
**Crot + 4-AP**	1.2 + 1.2	5’	Yes (~20 min)
**4-AP + Crot**	1.2 + 1.2	12’	Yes (~30 min)

On the other hand, the administration of voltage-dependent K^+^ channels blocker 4-AP (30 μg/animal, which corresponds to 1.2 mg/kg BW), before the *ip* injection of crotamine (1.2 mg/kg BW), significantly increased the time necessary for the onset of hind limb paralysis syndrome induced by crotamine, although it was not significantly changed by the administration of 4-AP after crotamine *ip* injection, neither this compound was able to protect the animal from death induced by crotamine, as also noticed for TTX injection (Table 1).

Interestingly, the administration of a steroid-derived plant alkaloid veratridine (VTD), which functions as a neurotoxin by abolishing the inactivation of Na^+^ ion channel, also promoted the hind limbs paralysis (S1 Fig), similarly as described for crotamine, although with no difference in the time elapsed for the paralysis onset depending on the doses of VTD administered (*i*.*e*. 1.5 or 6.5 mg/kg BW). In addition, no paralysis could be observed at dose as low as 0.5 mg/kg BW of VTD for continuous observation during up to 2 h. In addition, no animal death was registered up to 48 h after the injection of any doses of VTD assayed here (up to 6.5 mg/kg BW). On the other hand, *ip* injection of VTD (1.5 mg/kg BW) in animal that previously received a single administration of crotamine (30 μg/animal or 1.2 mg/kg BW) determined characteristic hind limbs paralysis as soon as 5 min after the *ip* injection of toxin, and the animal death was confirmed about 2 days after. Conversely, prior *ip* injection of VTD did not affect the hind limbs paralysis onset or animal death (which occurred about 40 min) determined by crotamine administered afterward (Table 1).

## Discussion

Snakebite envenoming by South American *Crotalus* rattlesnakes is mainly characterized by the rhabdomyolysis and neurotoxic effects [1,35]. Depending on the intensity or severity of the envenoming, other clinical symptoms as myalgia, myasthenic face and/or urine darkness may also be observed in the affected patient. Although the urine darkness, popularly described as ‘coke-like urine’, was first associated to possible hemolytic or kidney damage action of the toxins present in the rattlesnake venom, it is now recognized that in human, this is more likely determined by the myotoxic effects as rhabdomyolysis, which can eventually determine the kidney injuries [1,36]. All these effects have been mainly associated to the action of the neurotoxin crotoxin (phospholipase A2), also considered as the most lethal component of the rattlesnake venom (with estimated LD_50_ values between 60 and 180 μg/kg of BW in mice, depending on the administration route) [1,37,38]. Crotoxin is far more toxic than crotamine, which LD_50_ was described to be of about 0.07 to 6 mg/kg of BW, also depending on the administration route [5,7,8,9]. However, although significantly less toxic, crotamine may also contribute to the myotoxic effect as described by some researchers [9], as also confirmed by our present data, mainly for muscle spams and hind limbs paralysis.

Herein, we demonstrated for the first time a dose-dependent response for the hind limb paralysis that was characterized by a time delay in this response onset for lower doses of crotamine, but with no clear difference in the intensity or time of the immobilization effect. On the other hand, the voltage-activated K^+^ channels blocker, as the 4-aminopyridine (4-AP), was able to delay the hind limbs paralysis onset determined by crotamine, although only when administered before the crotamine injection (Table 1), suggesting a possible decrease in the potency of crotamine ability to determine the paralysis effect. Actually, this effect seemed paradoxal to us considering the effects of the presence 4-AP in the observed crotamine-induced increase of isolated diaphragm contraction force, as the potentiation of crotamine-induced increased contraction force by 4-AP was observed only when this ion channel blocker was added after crotamine stimulation (Fig 5). Blockers of voltage-activated K^+^ channels are able to prolong the motoneuron action potential, which thereby may increase the release of neurotransmitter at the neuromuscular junction, which led to their use as inducer of seizure in mice and for the symptomatic treatment in multiple sclerosis in human [39,40]. Interestingly, 4-AP was described to be capable of reversing the effects of tetrodotoxin (TTX) poisoning in animals [41], and both blockers seem to affect crotamine effects (Table 1).

This Na^+^ ion channel blocker TTX was also able to increase the time elapse for the hind limb paralysis induced by crotamine, but only when administered before the injection of crotamine (Table 1). Similarly, TTX was able to avoid the paralysis induced by veratridine (VTD), only when administered before the injection of this plant alkaloid (Table 1). On the other hand, the Ca^2+^-activated K^+^ channels blocker apamin (APA) did not influence this hind limb paralysis triggered by crotamine *in vivo*, which actually is in agreement with the lack of APA influence on the positive inotropic action of crotamine in *ex vivo* assays (Fig 5).

The importance of Na^+^ currents for skeletal muscle contraction is well known [42,43], and the selective stimulation of fast contraction of extensor digitorum longus (EDL) skeletal muscles, which was proposed to be positive to prevent skeletal muscle dysfunctions in rats with adjuvant-induced arthritis [44], was also described for crotamine [16]. Herein, VTD, which is a steroid-derived plant alkaloid that functions as a neurotoxin by abolishing the inactivation of Na^+^ ion channels [45,46], was found by us to be able to induce the hind limb paralysis similarly as described for crotamine, although with no observable time-response correlation with the injected doses (Table 1). In addition, differently from crotamine, this paralysis syndrome was blocked by previous administration of TTX and was not accompanied by the animal death. Interestingly, administration of VTD after crotamine injection delays the animal death, although the previous administration of VTD did not show any influence in crotamine effects on hind limbs paralysis onset or animal death (Table 1). On the other hand, VTD, which imposes persistent activation of Na^+^ channels, is able to protect the animal from death determined by the *ip* administration of TTX, either for pre or post-administration, but VTD does not protect animal from death determined by high doses of crotamine (Table 1). One hypothesis is that VTD would be leading to fading of tetanic tension imposed by high doses of crotamine *ip* administration, as VTD effect is localized to excitable sarcolemma, as confirmed by intracellular recording of action potentials, which also showed a marked VTD-induced fallout of action potentials during continuous 50 Hz stimulation, whereas endplate potentials were unaffected [15]. This all is in good agreement with previous report suggesting that besides some similarities (for instance the sensitization to K^+^ and direct action on muscle fibers), crotamine and VTD effects could not be due to the same mechanism of action [47].

The action of VTD on Na^+^ ion channels was also confirmed here by the *in vivo* assay showing that the previous administration of TTX can hamper the paralysis induced by VTD, although TTX cannot reverse the paralysis elicited by VTD if injected later, when the skeletal muscle is already immobilized (Table 1). Interestingly, the Na^+^ ion channels blocker TTX does not promote paralysis but does kill the animal, while VTD, which abolishes the inactivation of Na^+^ ion channel, promotes paralysis but does not kill the animals (Table 1). TTX and VTD affect crotamine-induced hind limb paralysis and animal death, respectively, and only when administered before and after crotamine injection, respectively. Although acting most probably by abolishing the inactivation of Na^+^ ion channels as VTD, crotamine promotes the skeletal muscle paralysis and also promotes the animals death at high doses, which is potentially protected by the prior administration of VTD or K^+^ channels blockers, but not by the Na^+^ ion channel blocker TTX (Table 1).

It is also possible to hypothesize that eventual intracellular effect of crotamine may occur in the muscle cells, as we have previously demonstrated for many other cell types that intracellular organelles, as the lysosomes and mitochondria, are potential targets for this toxin [48,49]. Actually, this intracellular action of crotamine driving to increases of intracellular free calcium (Ca^2+^) was the motivation to evaluate the eventual participation of the Ca^2+^-activated K^+^ channels, as APA, which ruled out the contribution of these channels in the effects triggered by crotamine on skeletal muscle contraction force (Fig 5).

We also hypothesize that the polysaccharide material that contacts the basement membrane of sarcolemma may also play a potential role in the effect observed for crotamine in skeletal muscle, as its affinity for proteoglycans present on cell membranes was largely studied and demonstrated by our group to explain the cytotoxic and antitumoral activity of crotamine [50,51,52]. In addition to its potential action on mitochondria, which are often numerous in striated skeletal muscle structure, the possible differential expression of proteoglycans in hind limbs and front paws skeletal muscles of an animal, may have equally the potential to explain the selective paralysis of mice hind limbs with no noticeable or important effects on mice front paws. However, further studies are undoubtedly still necessary to experimentally confirm this hypothesis. Interestingly, the fast contraction EDL muscle also presents lower oxidative capacity and mitochondrial density compared to the slow contraction muscle [53], and the effects of crotamine on mitochondria was subsequently demonstrated by us [51], although with no experimental demonstration to support the direct correlation between these findings up to now.

It is also important to consider that the activation of voltage-sensitive Na^+^ channels is often related with pain [54,55], being therefore the target for volatile anesthetics [56]. The voltage-gated K^+^ channels were also functionally related to neuropathic pain [57,58,59]. Interestingly, more recently, VTD was also reported to produce pain [60,61]. Considering that pain could interfere with normal animal behavior including the locomotor capacity, especially in the open field and sucrose splash tests, we also evaluated the eventual nociception for locally (intraplantal) and systemically (*ip* injection) administered crotamine, showing no observable signal of pain induced by crotamine (*ip*, 7.5 μg/animal), in a dose capable to determine the effective immobilization of mice hind limbs.

The open field test allows assessing the anxiety and the general levels of locomotion activity, and herein it was fundamental to show, for the first time, a dose-dependent hind limbs paralysis in mice, with a significant time delay for the paralysis onset at lower doses of crotamine, suggesting a inverse relationship for the crotamine dose versus time for paralysis onset of animal receving crotamine by *ip* route (Fig 7 and Table 1). In previous studies, the hind limbs paralysis was generally monitored only by naked eyes visual observation and transversally, *i*.*e*. during short periods of monitoring time [7,8], which could potentially explain the lack of reports of paralysis syndrome at lower concentrations of crotamine, showed here to occur later on, when the animals usually were no longer being monitored. However, even under this long term surveillance by video recording performed here, it was not possible to observe any possible gradative decrease of hind limb movements even with lower doses of crotamine than those previously reported to be necessary for the animal paralysis [7,62]. Interestingly, we finished observing only total paralysis onset (data in S2 Video), for any dose of crotamine evaluated, and the minimum concentration/doses of crotamine necessary for this effect could not be determined here. But at this point, it is important to consider that even after 21 days of daily *ip* treatment with crotamine (1 μg/animal, which correspond to about 0.04 mg/kg of BW), we have never observed the hind limbs paralysis or any other noticeable change in the general behavior of treated animals [50,52,63]. It is also worth to mention that the mice hind limbs paralysis was not observed up to 2 h after administration by oral route of a single dose of 200 μg of crotamine (which corresponds to about 8 mg/kg of BW), or after a long term daily treatment by oral gavage with crotamine (10 μg/animal, which correspond to about 0.04 mg/kg of BW) [52,63].

To our view, the sucrose splash test supports that the paralysis of the animal members induced by crotamine is restricted to the hind limbs, as the crotamine-treated mice could still perform the grooming behavior, although with a significant lower frequency along time (Fig 9). In addition, we could demonstrate that crotamine-treated animals were still moving the front paws if mechanically stimulated, even during the event of hind limbs paralysis elicited by the *ip* injected crotamine (data in S1 Video). As mentioned, the grooming process was effectively decreased in crotamine-treated animals compared to the control, but probably due to other factors that do not seem to be related to the paralysis or difficulty of moving the front paws. In light of these observations, the possible effect of pain was then considered.

Further studies are still necessary to explain this selective action of crotamine for the hind limbs skeletal muscle, as this particular and unique action of crotamine on the skeletal muscle ion channels described for the first time here, unfortunately, may not explain alone this selective effect on hind limbs, since up to our knowledge there is no specific difference in the expression or function of these ion channels in front paws and hind limbs skeletal muscles of mice.

Interestingly, mouse strains that lack the expression of mechanically activated nonselective cation channels present severely uncoordinated body movements and abnormal limb positions [64]. We noticed that knock-out mice for this specific mechanotransduction channel for proprioception phenotype is very similar to the paralysis syndrome described by us here for crotamine, mainly characterized by the hind limbs paralysis with no alteration of the movements of the front paws (data in S1 Video). Therefore, the mechanisms involving this channel should also be potentially considered to explain the selective action of crotamine in the mice hind limbs, and it my further explored in our next studies.

Cancer patients under chemotherapy treatment experience decreased physical fitness, muscle weakness and fatigue, including under therapy with drugs as docetaxel, which is commonly used to treat cancer [65]. Although intravenously administered docetaxel does not impair the force production in hind limb muscles in healthy mice, a tendency to decrease the peak force is often observed in soleus muscles, 24 h after a single injection of this drug (-17%, *p* = 0.13), and this is often used to explain the side effects associated with chemotherapy (neurotoxicity, central fatigue, decreased physical activity, etc.), which is described as experienced muscle weakness and fatigue [65]. Therefore, the increases in the force contraction of skeletal muscles promoted by native crotamine at very low doses, once associated with current chemotherapy drugs might have the potential to decrease this specific side effects with an eventual potentiation of the antitumoral effects of both compounds, although we are conscious that experimental studies to specifically confirm this hypothesis are still necessary.

We also hypothesize that the antitumoral and even the antifungal effect described for crotamine [52,62,66] could potentially involve the K^+^ channels, and the contribution of these ion channels for cancer and antitumor treatment efficacy is increasingly recognized [67,68,69,70]. In addition, at this point, it is worth to mention that under the conditions employed for the antitumoral treatment with native crotamine, by *ip* (1 μg/animal/daily) or oral (10 μg/animal/daily) route during 21 days, we never observed any sign of skeletal muscle paralysis or any other detectable toxic effect under the conditions employed up to now [50,52], even during long-term treatments of healthy animals [63], which is very supportive to our continuous effort to translate this molecule to clinical use.

Unfortunately, at the moment, it was not possible to identify which factors mainly determine this selective immobilization of the mice hind limbs, with no important effect on their front paws. New studies are already being planned to clarify this aspect. Althougth the well-known and characteristic myastenic face and other myotoxic effects of rattlesnakebite envonoming could not be attributed to crotamine action, we could demonstrate that the purified crotamine promotes positive inotropic effect of isolated skeletal muscle with some observable signs of myolysis, and can also kill the animal when injected acutelly at high concentrations.

More importantly, involvement of the Na^+^ and K^+^ channels in the hind limb paralysis syndrome triggered by crotamine in mice was confirmed. In addition, the concentration-dependent ability of crotamine to potentiate the contraction force of isolated skeletal muscle, which was intensified by the voltage-activated K^+^ channels blocker 4-AP, but not by the selective blocker of Ca^2+^-activated K^+^ channels APA, could be clearly demonstrated here. Similar and convergent results were also observed by employing *in vivo* assays, and therefore we suggest that the voltage-activated K^+^ channels are also involved in the rigid immobilization effects of crotamine in skeletal muscle of mice. As the selective inhibition of the Kv_1.1_, Kv_1.2_ and Kv_1.3_ channels by crotamine was reported [20], further studies aiming to identify the subtypes of voltage-activated K^+^ channels involved in crotamine induced skeletal muscle paralysis effects that could explain the selective immobilization of the hind limbs skeletal muscles will be the matter of our next study.

As we have described the ability of crotamine to elevate the levels of intracellular free Ca^2+^ [51], one could also expect that the effects of crotamine in the muscle contraction could involve the opening of Ca^2+^-activated K^+^ channels, since in any skeletal muscle cell, increased intracellular Ca^2+^ causes contraction, and a dynamic relationship between Ca^2+^ transients and force production was described in intact muscle fibers under physiological conditions [71]. However, as mentioned, the selective blocker of Ca^2+^-activated K^+^ channels, namely APA or the scorpion toxin CBTx (Fig 5) did not significantly change the skeletal muscle contraction force elicited by crotamine on isolated diaphragm, neither APA showed any effect on hind limb paralysis induced by intraperitoneal (*ip*) administration of crotamine (Table 1), which in our view preclude the participation of members of this K^+^ channels family in the crotamine characteristic paralysis syndrome.

Also, the synergism among the different toxins present in South American rattlesnakes should be also considered, as mainly due to the relative low complex composition of their venom, enhanced pharmacological injuries would be desirable to increase their chance to capture preys and assure their survival in the nature. In fact, the synergistic mechanism of toxicity is progressively more accepted and studied in many envenoming processes determined by several venomous animals [72,73], and this might also be a potential matter of study to better clarify the role of crotamine on envenoming by *Crotalus* snakes.

Finally, although the previous administration of VTD (a compound that abolishes the inactivation of Na^+^ ion channels) before the TTX injection could protect the animal from death induced by TTX, VTD was not able to protect the animal from death induced by crotamine (Table 1). Therefore, further studies are still necessary to clarify the mechanism(s) underlying crotamine contributions in animal death, but the present data have the potential to contribute to ameliorate the therapeutic interventions and clinical outcomes of patients victimized by snakebite envenoming by *Crotalus* genera rattlesnakes containing crotamine in their venom.

## Supporting information

S1 Video Tail suspension of mouse with typical hind limb paralysis after crotamine administration. (M4V)

S2 Video Typical hind limb paralysis after *in vivo* administration of crotamine in mouse. (MP4)

S1 Fig Hind limb paralysis of mouse after crotamine administration. (TIF)
