# Supplementary figures and images for: Pharmacological characterization of crotamine effects on mice hind limb paralysis employing both ex vivo and in vivo assays: Insights into the involvement of voltage-gated ion channels in the crotamine action on skeletal muscles
Source: PLoS Negl Trop Dis. 2018 Aug 6;12(8):e0006700. doi: 10.1371/journal.pntd.0006700 (PMC6095621; doi:10.1371/journal.pntd.0006700)

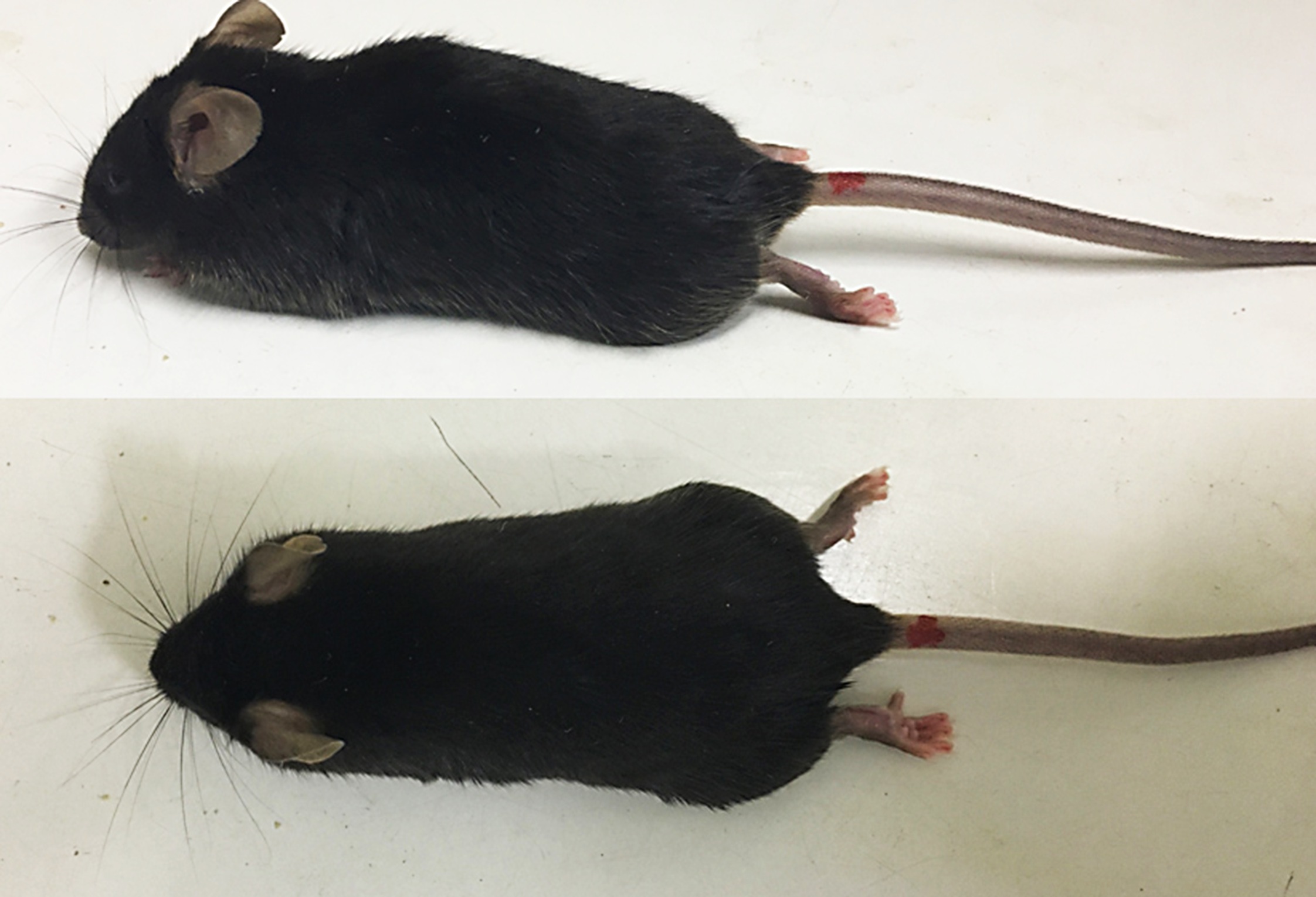

Supplement: S1 Fig — (TIF) [file pntd.0006700.s003.tif]
